# Triune Man.—The Inaugural Discourse Delivered at the Opening of the Last Session of the London Hospital Medical College

**Published:** 1856-10-01

**Authors:** Andrew Clark


### Art. VII.—TRIUNE MAN.

### THE INAUGURAL DISCOURSE* DELIVERED AT THE OPENING OF THE LAST SESSION OF THE LONDON HOSPITAL MEDICAL COLLEGE.

BY ANDREW CLARK, M.D.

"And God† said, let US make man in our image and after OUR likeness."
Genesis, c. i. v. 26.

"The invisible things of Him from the creation of the world are clearly seen, being understood by the things that are made."—Romans, c. i. v. 20.

GENTLEMEN,—It is a great occasion which calls us together to-day. Fraught with the deepest interests of individuals, and through them of humanity, its importance cannot be overrated, nor its objects too zealously enforced. It is the starting point of one of those great journeys which, collectively, constitute the business of existence, and the design and conduct of which will not only determine for ourselves the final failure or success of the end for which we live, but will reflect upon others also the good or the evil which must arise from duties neglected or fulfilled.

It is an occasion which may prove distasteful to the unthinking, frivolous, and idle; but it is one which must prove peculiarly welcome to the earnest in object, the heroic in self-denial, the resolute in will—to all those who are learning to feel and to know that man is born into the world to do something else than to eat, and to sleep, and to drink, and to die; that life is not an arena of pleasure, but a field of battle; that the end of existence is not the fullest enjoyment of its sensuous attributes, but the completest development of all that is true, and therefore divine, in man.

We meet, gentlemen, to inaugurate the business of another academic year; to cast another stone upon that cairn which has been raised by the long labours of those who have gone before, which stands behind us now the lasting memorial of all that we venerate within these walls, and which speaks to us in tones

* This discourse is printed, with some verbal alterations, as it was delivered. It is published reluctantly, and only at the renewed entreaties of several correspondents. It was written under unfavourable circumstances, and within a few days of its delivery. It is inadequate to the importance of the subject with which it deals: it leaves undeveloped the theory of man which it promulgates; and fails to define the nature and limits of the relations which that theory holds to the science and art of medicine. I have on these points, however, sacrificed my own opinion to that of others, in the earnest hope that the Discourse may suggest something of what it fails to convey.

† In this and several other passages of Scripture the substantive here translated "God," occurs in the original in the plural form, and might be rendered "Gods," or, more literally, the "adorable ones." Hence, (c. iii. v. 22,) "God said, behold, the man has become as one of US."

inarticulate, but felt, of what will touch every hopeful heart to-day—of many a past beginning and its end—of lofty resolve and base expediency—of tedious self-denial and low indulgence—of earnest effort and vacillating desire—of ardent struggle and cowardly compromise—of glorious triumph and merciless but deserved defeat.

Present wisdom is often but the reflexion of past teachings transmitted through new conditions. Humanity is ever the same, though the circumstances under which it manifests itself may differ. It is ever repeating, renewing, recombining, reproducing. And so to-day we are met—like those who have met here before for the same purpose—to begin *our* new work, which, like theirs of the old time, is fraught with the same dangers, pregnant with the same promise, and stakes upon its success all that is true—otherwise all that is desirable, worthy, or great in life. Now if we would learn how to shun the rocks and quicksands upon which others have been wrecked or broken into useless fragments; if we would learn how to realize something more of the possible glory of humanity than the compass of mercenary ends, the enjoyment of unsubstantial pleasures, or the miserable consciousness of an unmerited repute; if we would know how to fulfil God's purpose in our creation, and to become justified at the close of life before God and our fellow men, we shall not only listen to those inarticulate histories which now hopefully and mournfully come back upon us from the past, but we shall pause, and at the outset of this business which we are about to undertake, we shall solemnly, reverently, with purged hearts and earnest minds, address to ourselves these questions:—What *is* this work which we have before us to do? What are the instruments of this work? How are we *rightly* to do it?

In addressing myself to the consideration of these questions, and attempting such a rough reply to them as the occasion will permit, I undertake a task of no common difficulty. There are assembled here to-day the young and inexperienced, to whom knowledge is an opening mystery and a strong desire; the student who has already penetrated somewhat into the depths of things, and has begun to speculate about their meanings and relations; the gifted contemporary with his abundant knowledge and ripe reason, casting about for undeveloped truth; and the elders and fathers among us to whom knowledge and experience have been in some sort realized and consummated, and from whose lips I would gladly learn wisdom. One has his sanguine dreams, another his prophet-oracle of materialism. Some have their eclectics and their reasoned theory; others their rooted dogmas, their settled systems, and their method.

The object of all may be the same, but each has his different medium of vision and point of interest; each his own peculiar standard of judgment, and veracious,* or veritable test of success. To combine these conflicting elements into one, by enlisting sympathies and appealing to interests at once great in themselves and common to all, and to break ground upon a subject which constitutes the underlying greatness of humanity, the everlasting inheritance of individual men, and the medium of that perfect insight through which we are "to know even as we are known," is an attempt which needs, and for which I crave, your most generous indulgence.

In considering and answering the great questions which we have preferred to ourselves for solution, we shall have to go somewhat out of the beaten track pursued on such occasions, to lead you into what will prove to many new and untrodden fields of thought, to bring to light the fallacies and dangers of that gross materialism which pervades and pollutes the current of modern thought, and to lift up our voice, feeble as it is, in support of that view of the spiritual constitution of man which is being rapidly engulphed in the whirlpools of a drivelling and insensate reason. Questions of this kind lie at the root of all knowledge, all being, all life; and, for these to be sound, their foundations must be sure. I cannot apologise, therefore, for leading you into discussions upon the issues of which so much is staked. If my arguments should carry no conviction with them, and the hypothesis I advocate be declared untrue, they will not be valueless; they will provoke you to reflection, they will call forth your higher faculties into exercise, and they will make you think about the truth and seek it. However imperfectly these views may be embodied, I am in earnest about them, and if I fail in impressing you with their truth, I shall yet believe it is from no imperfection in the subject, but from impotence in the speaker.

The burden of the business in which we are about to engage is knowledge; and that our subsequent reflections may be endowed with unity and coherence, I shall speak to you in the first place of knowledge in general; and in the second, of that particular kind of knowledge which in this College it is *our* privilege to communicate and yours to acquire.

To comprehend the full significance of knowledge,—to perceive

* A friend, with the best intentions, informed me at the close of the delivery of this lecture, that these terms were synonymous. I apprehend, however, that the one has a *very* different meaning from the other. *Veracity* is the correspondence between the assertion of a man and his conviction, opinion, or belief. *Truth* is the correspondence between the assertion of a man and the absolute reality or fact. A statement may be *veracious* (*i. e.* believed by the maker of it to be true) and yet not veritable—*i. e. true.*

the relations whereby it links manhood with Godhood—finitude with infinity; to realize its intrinsic worth and glory, to appreciate in harmonious concord and balance its spiritual and material utilities, and to feel it in our hearts as a divine commandment, a necessary means of development, and in its highest sense the object of life,—we must possess some definite conception of the constitution of man, who is the subject of knowledge—its sovereign and yet its slave.

But a knowledge of the human constitution is necessary fo[illegible] other reasons than this,—for graver and greater ones than its being merely the highest gymnastic of the mind, and the noblest means of its development and growth.

In the exercise of our calling, it is impossible for us to deal knowingly and wisely with the various disorders of the animal body without distinctly recognising the agency of states and conditions of mind, often in producing, and always in modifying them when produced. There is, indeed, a very intimate relation between the moral and material elements of the human constitution. They act and react upon each other in modes more numerous and varied than is yet known or even conceived; and out of their mutual motions develop phenomena, many of which remain as inexplicable as they promise to prove important.* The broad fact of this relation is everywhere admitted, and has become a household word. Special manifestations of it occurring in the daily business and intercourse of life are familiar to the feeling and perception of every thinking man. Not only do general states of mind produce corresponding conditions of body, but it seems certain, from recent and carefully-conducted experience, that the concentration of the intellect upon particular parts of the animal frame is capable, within certain limits, of effecting such a change in them as the will may determine. A large and growing series of facts of this character has already been elicited; but from our ignorance or indifference, our prejudice or unbelief, they remain undigested and unclassified—vague subjects of idle or vicious speculation, and altogether useless in their relations to the practical business of life. With a liberal allowance for much in these alleged facts that is either exaggerated, imaginary, or false, enough remains to excite our most earnest curiosity, and to justify a deliberate and systematic inquiry into their claims upon our belief.

But I have further to observe, that the very admission of the existence of these relations renders it a sacred duty on our parts

* Dr. Carpenter's later papers, however, throw much additional light on this subject. They are full of profound and ingenious thought, and display a remarkable aptitude in the writer for the investigation of this very difficult department of truth.

to investigate them—to attempt the discovery of the laws under which they are manifested, and to render them practically subservient, if that be possible, to the relief of human suffering and sorrow. There is nothing improbable in the supposition that such an investigation might lead to the development of a system of *moral therapeutics* as valuable as an element of treatment in chronic, as material therapeutics are in the treatment of acute disease. If it led to nothing else than the more successful comprehension and control of that vast variety of functional disorders so inseparably allied with advanced civilization, and which form such frequent and serious hindrances to the duties and enjoyments of life, it would prove a benefit of no common value to the race. View the question, however, as we will, dubious as we may be of its ever becoming realized into a system of practical utility, it remains for us to remember that whatever is real and true in these relations should not be lost to legitimate medicine—that it should be rescued from the hands of impostors and quacks, who have perverted the knowledge of these relations to the vilest of purposes, and made it at once a religious abomination and a moral pest.

For these reasons, then—that you may comprehend the true significance of knowledge, and realize the true object of its acquisition, and that you may be enabled more efficiently to exercise the noble profession to which you are called,—it is necessary that you should possess some knowledge of the mental as well as the bodily constitution of man. Using the term in its narrowest sense, an ancient writer has said, with great shrewdness and no little truth, that philosophy should end with medicine, and that medicine should begin with philosophy.

The universe, says the modern philosopher, is composed of mind and matter. Man, the microcosm—the universe within the universe—is composed of soul and body. Knowledge, therefore, is of two kinds—that which pertains to matter, physics, or natural philosophy; and that which pertains to soul or mind, metaphysics or moral philosophy. This is a classification at once plausible, practical, and plain. It may be readily realized by the meanest capacity. It attracts the methodical by its broad distinctions, and by the elasticity through which it becomes capable of such ingenious adaptations to the varying aspects of things. The pursuit of knowledge under its auspices has conferred incalculable material benefits upon the race. Out of barbarians it has developed men. Of man it has made almost a God. It has placed the powers of nature under the control of his will, and rendered them subservient to his enjoyments and uses. But, for all this, it is not perfect. In relation to man, it s not even true. For, high as man stands in the scale of being—great as have been his achievements in his conflicts with nature for knowledge and power—his position is not so high, nor are his achievements so great as they surely might have been if, with clearer views of the human constitution and more earnest efforts for the realization of his destiny, he had addressed himself with all the powers and all the purity of his nature to the task of subjugating the unnecessary accidents of his time and being, and struggled through legend and prejudice to stand face to face with the unveiled glory of immaculate truth.

In relation to man, this dual view of the human constitution is imperfect, because its results are altogether onesided and incomplete, and because it wholly consults his material at the expense of his spiritual wants. It is *untrue*, because, pursued into its logical consequences, it ignores a spiritual element in the human constitution, resolves man into a mere unity of material organization, attributes that organization to an ordinary combination of elements common to the inorganic world, and solves the problems of organization and life, of morality and religion, by the declaration of an unguided history and the enunciation of a lawless law.

Admit that man has a dual constitution—that he consists of what is called mind and matter—and let us look at the logical consequences of this admission. Mind is known only by its manifestations. Where sensation, perception, judgment, reason, memory, *are*, there mind *is*. These properties or faculties are found in man, and in him we say there is mind. But they are also found elsewhere. We cannot investigate the psychical phenomena of inferior animals without being forced to admit that, over and above what we call instinct, there are to be found sensation, perception, intelligence, reason. In the lowest forms of animal life we find indications only of sensation; but that is a property correlative with the others, and an attribute of one subject. The difference is one of *degree* only, not of *kind*. The psychical quality seems in the last degree clearly referable to the physical organization. Yet *there*, according to our reasoning, is *mind*. But in the lowest forms of animal existence, both reason and revelation—if that be admitted—oppose the admission of an immaterial principle. Philosophy forbids the introduction of a second cause of things where the first is sufficient. *Entia non sunt multiplicanda præter necessitatem.* Mind, therefore, comes to be the mere attribute of matter, sensation a peculiar property of the nerves, and thought as much a secretion of the brain as bile is of the liver. Hence, by an inevitable logical necessity, which it is at present unnecessary for me to develop or demonstrate, has arisen that system of sensationalism which has culminated in the boasted Positive Philosophy of Auguste

Comte—a system which reduces our mental operations to forms of sensation, morals, to the calculations of self-interest and expediency, and religion to an old wife's fable;—a system which ascribes all terrestrial phenomena to the spontaneous evolution of blind mechanical laws; which professes to demonstrate how man with his present knowledge could have designed the world with more excellent purpose and skill; which resolves man into a mere automaton, and his hope of immortality into a delirious dream; which turns God into a figment of the fevered fancy, and this glorious universe into a sorry system of self-sustained machines.

Such views are not only repugnant to the innate instincts of humanity, but can readily be refuted by reason, and—as I am addressing a Christian audience—by revelation. There is truly no evil without its counterbalancing good; and the shocking conclusions forced upon his acceptance by such a system as this has at last aroused man from his deadly lethargy—urged him to more earnest inquiry about humanity, nature, and God, and compelled him to seek some substantial foundation for his irresistible faith.

Thus we see that dualism ends in unitalism, and that unitalism makes man merely a common combination of common elements.

But this apprehension of the direful dangers of dualism to the dignity of humanity is no novel product of modern thought. It is but in some sort a revival of that subtle intuition which in ancient days impelled every true thinker to struggle after some loftier conception of the constitution of man, and bravely, self-denyingly—often fiercely, to compass all the circuits of imagination and reason for the discovery of the sacred truth which lay embosomed in their depths.

From Diogenes to Plato the solution of this problem was the life-struggle of many an earnest soul yearning in deep desire to realize the divinity of man. Often, through the unsettled mists of uncertainty and error, they caught scattered glimpses of the truth. Often were they led to the very threshold of the sanctuary in which she dwelt, and only paused before the veil because they knew not where or how to lift it up. And though at last they failed to penetrate the garnitures of this mighty mystery, or rather to prove that they had penetrated them, they wavered not in faith. They felt, indeed, that they *had* probed the mystery, and that, in their own consciousness at least, they had even realized its solution. But they felt also that they had failed to reason this realization into logical form, and that in struggling after the grounds of certitude they brought to light only the foundations of doubt. And so, at once mournfully and and yet hopefully, they appealed to their irresistible belief, and left their divine inheritance for posterity to vindicate and enjoy.

There is something inexpressibly noble in the efforts of these great minds to solve this great problem ; something inexpressibly touching in the confessions of their incompetency ; something, too, inexpressibly delightful to us in the admission, that though they did not furnish us with the workings of the problem, they have handed down to us, and all that are to follow after us, the real results of its solution.

In the productions of all the master-minds of ancient philosophy, there is to be found an uniform tendency to attribute a tripartite instead of a dual constitution to human nature.

Strabo and Arrian tell us that the Gymnosophists, or Brachmans, taught the threefold nature of man. If we examine the writings of Pythagoras, Plato, and Aristotle, we shall find that each asserts, though in different terms, the same expression of belief. The last, indeed, not only touches the truth, but probes it to its very centre. He fails only in reasoning it. Man, says Aristotle, is composed of three principles: the *nutritive*, the *sensitive*, and the *rational*. The first principle is that by which life is produced and preserved ; the second, that by which we perceive and feel ; the third, that by which we reason and feel.

So, among the latter Jewish and earlier Christian authorities, we find a similar theory of the human constitution enunciated and enforced.

In the Jewish Catechism, in the Talmudic treatise "Maccoth," and in the canonical books of Proverbs and Ecclesiastes, it is taught, that man consists of three elements: Nepesh, Neschamah, and Teschidah. Nepesh is life ; Neschamah, intelligence ; and Teschidah, the spirit or divine principle by which man may reach unto God, and become identified with the ensophic world.

Among the early Christians, too, the same theory was universally propounded and received. Saint Paul repeatedly speaks of the threefold nature of man ; and in a solemn prayer for the Thessalonians, he expresses the hope that their spirits, their minds, and their bodies should be preserved blameless unto the coming of the Lord. And if the reasonings of Jowett should nullify the authority of Paul, God himself has told us, by a hitherto unquestioned tongue, that He made man after His own image.

Here, then, is a theory of the human constitution which has been propounded by the greatest men of all ages, and of all dynasties, whether pagan or Christian ; a theory which, though unreasoned, has yet been received and believed by the thoughtful and earnest among men, because it satisfies the instinctive cravings of humanity, and vindicates the divinity which is felt to be its divine inheritance and its right; a theory which has for its only rival a system wholly gratuitous in its foundations, confessedly imperfect in its objects, and absolutely revolting in its results.

Against the conclusion from these accumulated ages of reason and this direct revelation, our modern philosopher opposes his dual view of the constitution of man; and in attempting to substantiate it by reasoning it, lands himself at last in the mires of a solitary materialism and infidelity.

We have no consciousness, says our philosopher, but that which is supplied to us by the successive operations of the mind. These, collectively, constitute the individual. On this single premiss I pin my faith. The conclusions to which it leads are decisive in destroying your hypothesis and establishing my truth. True, O philosopher, if we admit your premiss. But *is* your premiss *true?* I am not quite sure about that. Let us look at it a little more closely.

If we have no cognition of self other than in the changes which self undergoes, we can have no possible knowledge of the operative causes of those changes. Personality is lost; the spontaneous will ceases to be a living fact; the active intellect becomes a dead machine, and man only a blind, insignificant puppet, moved by the strings of accidental circumstances, coming he knows not whence, acting he knows not how, going he knows not whither—away into the dark, unconscious and unseen.

Why should I go farther? Is not this logical consequence of the dual theory enough to typify the rest? Are you prepared to accept them? Are you ready to act upon and abide by them for ever—to make them the bounding lines of your life, hope, joy, and destiny? Is this really, then, to be the consummation of all our thinkings and actings—of all our battles and victories—of all our triumphs over life, nature, and ourselves—of all our instinctive and irrepressible longings after the permanent and divine of this fleeting and visionary world? Are we to find in such a theory as this the foundation of all our exalted notions of right, duty, virtue, religion, love? Does irresponsible and irresistible response to excitements constitute the boasted glory of humanity? Is this gross and passive mechanism, called man, all that is to spring out of that Divine Breath which of erst was breathed into it by the Omniscient, who is said to have made it like unto Himself, a *living soul?*

Thank God, it is not so. For the sake of our common race and our common destiny, we rejoice to believe that there is something more in man than the earth—earthy; something nobler than blindly responsive matter; something spontaneous

—something divine—something which lifts him far above the passive existences of the universe, and links him indissolubly with God, who became incarnate in his frame.

When I think, I am conscious not only of the thoughts present to the mind, but also of the self which is thinking, and to which the thoughts are present. I distinguish the thought as object, and the self as subject. And though sensations, perceptions, and memories do of themselves constitute a sort of running consciousness which cannot be separated from them, there is to every man a consciousness over, above, and independent of them. This over-consciousness, so to name it, is the absolute *ego*—the self—the region of personality, spontaneity, and abstract reason—the thirdfold element of man—the abode and sanctuary of the spirit. Do not suppose I hold any or all of these to *be* the spirit. What the spirit is, I neither know nor conceive. I speak of these as the organs of the spirit, of which the will, perhaps, is chief. The spirit has profound thoughts, deep insights, divine impulses, but no language. The subjects of its consciousness are incapable of investiture in words. They can be felt and realized, but not expressed except through the manifestations of the will and in its control.

Let us to the logical proof of all this—that proof which we covet when we cannot get, and despise when gotten.

1. Matter cannot become known unless in union with mind. Mind cannot become known unless in union either with matter on the one hand, or self on the other. But we are still conscious of self above these. This self is spirit; and so spirit becomes known to us of itself unto itself—unconditioned and alone. But spirit is unconditioned only in reference to man. In obedience to the universal law, and to complete the scale of Intelligence and Power, spirit must be posited by something higher than itself—something in which it must become realized and known. So spirit, so too all possible spirits become objects to God, who is at once their eternal subject, absolute substratum, and everlasting source.

2. Again, every possible cognition implies a synthesis of subject and object. But the mind reflects upon its own operations, and has knowledge of them. It is certain, however, that in such reflections the mind cannot at one and the same time be both subject and object, the synthesis of which *alone* constitutes knowledge. Here, then, mind is the object. But it must be the object of some subject. That subject is the spirit. Spirit, then, is the thirdfold element of man—that Divine Breath which links him on the one side with the universe, and on the other with God.

The spirit is little influenced by impressions from without, and only by those which are real. The gaudy shows of the outer world, our routine reflections, our compensating expediencies for violated laws, and our ostentatious philanthropies, hang like cumbrous clouds around the spirit, shut it out from the mind, and force it back into its unfathomed sanctuary, to brood and beget there unheeded and unheard. It is in the solemn silence of night, in the abnegation of barren theories, and under the influence of earnest aspirations for the realization of God in ourselves and in the world, that its silent but significant workings become manifested to us—that the barrier between God's grace and man's capacities is cast down, and that the divine light rays out of the abyss through our minds upon nature, and guides us unerringly to the conquest of her mysteries. It is from the spirit, stripped of adventitious garnitures, that there has sprung all that is great, and true, and beautiful, and holy in art, poesy, and religion. From the spirit must arise the greater glories of the future, like incense from a censer lighted by the breath of the Almighty, and fanned by the purified affections of man. Through the spirit alone can we rise to the knowledge of God, and hold with him that holy communion which is the natural privilege and function of every disciple of Christ. Only through the spirit can we become one with Christ, and the sons of God.* Spirit, then, is manifested to man, not from beneath, but from above: it rays into his consciousness like an ever-living light and glory from the hidden but acknowledged unseen: it is deeply felt amid the shows and insincerities of social and personal life, and realized under earnestness and faith: it is the immediate, though as yet imperfect, revelation to man of the Eternal Image in which he was created, and towards which, by this very community of nature, he feels himself forced to struggle irresistibly, and yet consciously, for evermore.

But there are other and higher reasons than merely logical ones for upholding the tripartite constitution of man: for in whatever light we examine the characters of this wonderful being—naturally, historically, or theologically—we find equal evidence of his threefold nature.

Naturally, the threefold man is indicated in the normal development of his being.

In infancy, there is the incessant energy of animal life, the play of instinct, and the force of habit. Physical organization is supreme.

In youth, obedience to instinct is replaced by sensation and intelligent desire: the exuberance of organization is restrained by the development of mind: sensation and its products sway.

In the matured man, instincts, sensations, and desires are

* The natural (ψυχικός) receiveth not the things of the Spirit of God; neither can he know them, for they are spiritually (πνευματικῶς) discerned.

rendered subservient to the spirit, which manifests itself mainly through the consciousness and will.

The individual is the archetype of the nation. Historically, it will be found that nations have passed through the same phases of development as the individuals who constitute them.

Theologically, man peculiarly displays the proofs of his own constitution.

It was through the medium of the senses that God first communicated with man. This was the Patriarchal age.

In the succeeding era of human history, God communicated with man through the mind. This was the period which began with palmy Egypt, and terminated in fallen Greece.

In the final stage of man's theological development, God addressed Himself to the spirit of human nature, through the incarnation of the Word.

In the Patriarchal dispensation, we have merely material sacrifice; in the times of Law, revelation is written and addressed to the reason of humanity; in the age of Christianity, the *spirit* of man is spoken to through regeneration and the gift of the Holy Ghost.

In the next place, I would observe (and the observation is a very solemn one), that unless we admit the threefold nature of man, we cannot comprehend that which is the ground of every Christian's faith—the perfect manhood and Godhood of Christ. Christ had perfect knowledge of God—was God and yet man. He had a human body—life and all its sufferings. He had, however, a finite mind, for we are told that he *grew* in wisdom and knowledge. But He had a perfect spirit, for He was God, and from first to last grace or spirit-full. Man, too, had the elements of perfection; but the spirit was scant and soiled. So Christ suffered for humanity, and justified it. He had all, and was all.

Finally, if there is no spirit, there is no revelation. "Religion is the revelation of a spirit to a spirit." The natural man understands it not: it is superstition, ignorance, or folly, to him. Sensation and intelligence, which are the natural parts of man, enable him to investigate phenomena, to discover *relative* truth, to develop laws, to upbuild science. When properly cultivated, they enable him also to reap delight from the varying aspects of nature and the revelations of genius—from forms of beauty, melodies of colour, harmonies of sound—and from all that is great and true in the poet's song, or the chiselled embodiment of the sculptor's dream.

But all this is finite, physical—earthy. The means of its discovery is sensation; the only test of its truth, experiment; its highest conceivable consummation, law. But absolute truth is not to be thus discovered. The truths of the spiritual world, and our relations to it, and to the world in which we yet live and have our being, are not to be evolved by seeing or touching, nor exhausted by the expression of a universal law. Out of the abyss of his own soul, every man, at some time, receives startling intimations of this truth; but the clouds and mists of over-cultivated sensations, which hang like a thick darkness round the soul, too often prevent them from becoming operative in their Divine design. Truly, eye hath not seen and ear hath not heard the blessed truths which the Creator communicates to his creatures. Who by searching can find out God?

*Reason*, as well as *revelation*, therefore, demands our acknowledgment of a tripartite constitution in man. Man possesses life in common with plants, mind in common with animals, spirit in common only with God. Life, mind, and spirit are the threefold elements of the living world. Imperfect apart—perfect joined—individually manifested in plants and animals, they are combined and consummated only in man. He, like a well-ordered State, exhibits for our consideration,

1. The Spirit—the sovereign power.
2. The Mind—the deliberative council.
3. The Life—the subordinate executive.

Man, the centre of connexion between the universe on the one hand, and the Deity on the other, is, like each, a trinity in unity—the threefold perfected in the one.

Body, mind, and spirit are God's loans to man, which he is required to put out to use, and to return with interest. Life is a probation period; its end, eduction, development, or, as it is commonly called, education. The means of this education are three:—faith, grace, and will, for the spirit; sensation, perception, and knowledge, for the mind; food, labour, and law, for the body. The due development of these elements in their proper and fixed relations to each other, constitutes the harmonious unity of man. Excessive eduction of one part, at the expense of another, disturbs the balance of the whole, develops discord, and determines disease. Perversion of the bodily development makes the sensualist; of the mental development, the infidel; of the spiritual development, the fanatic and the mystic. The fluctuation of these elements throughout the past ages of the world have become the landmarks of human retrogression and progress. Like a wave of the sea in alternate ebb and flow, sensation now swells into scepticism, and faith subsides into superstition. Life, in all its present aspects, is but the battle of one-sided developments—the blind and blinding struggle of one element for supremacy over the other. Hence the loss of catholic unity, and the dogmatisms of modern socialists and sceptics; hence the virulence and bigotry of religious sectaries; hence our party politics, petty philanthropies, poorhouses, penitentiaries, and prisons—

man's attempted but vain compensations for the wilful violation of eternal laws. Solemn and weighty thoughts these, which it were well for all of us to meditate upon, but which, I remember, it is neither my province nor privilege at this time to discuss.

From this one-sided development of the elements of humanity, too, have sprung other evils far more melancholy and deplorable than even those of the grossest materialism and the most rampant infidelity. The logical consequences of the Positive Philosophy have proved so repugnant to the native instincts of humanity, that some minds—even earnest and true ones—protesting against their validity, and searching eagerly for the grounds of their refutation, have been carried away by the force of their instinctive beliefs, and have rushed madly into the opposite extreme of sacrilege and superstition. The reaction from materialism drowns itself in mysticism, and has developed a spirit which, manifesting itself in the unhealthy forms of mesmerism, electro-biology, table-turning, spirit-rapping, and other inane, false, partial, and exaggerated developments of man, "makes familiar play of the holiest instincts of humanity, and barters our firm beliefs and our righteous reverence for the sickly aberration of perverse and prurient imaginations." This reaction has revived the blasphemous idolatries of human power, and evolved a spirit which, arrogating to itself the power of a God, "yet gropes for the very holy of holies in kennels running deep with the most senseless and God-abandoned abominations."* "Our natural superstitions are bad enough; but thus to make a systematic business of fatuity and profanity, and to imagine that we are touching the spiritual kingdom of God and controlling it, is inexpressibly revolting and terrible. The horror and disgrace of such proceedings as those of the mesmerist and spirit-rappist were never approached even in the darkest days of heathendom and idolatry. Oh, ye who make shattered nerves and depraved sensations the interpreters of truth, the keys which shall unlock the gates of heaven, and open up the secrets of futurity;—ye who inaugurate disease as the prophet of all wisdom—who make sin, death, and the devil the lords paramount of creation;—have ye bethought yourselves of the downward course which you are running into the pit of the bestial and abhorred? Oh, ye miserable mystics, when will ye know that all God's truths and all man's blessings lie in the broad health, in the trodden ways and in the laughing sunshine of the universe; and that all intellect, all genius, all worth, is merely the power of seeing the loftiest wonders in the commonest things?"

* Quoted through memory, with alterations, from Ferriar, the distinguished and eloquent author of "Knowing and Being."

(*To be continued.*)

